# Analysis of Endocrine Disrupting Nonylphenols in Foods by Gas Chromatography-Mass Spectrometry

**DOI:** 10.3390/foods12020269

**Published:** 2023-01-06

**Authors:** Sang Mi Lee, Daeun Cheong, Meehye Kim, Young-Suk Kim

**Affiliations:** 1 Department of Food and Nutrition, Inha University, Incheon 22212, Republic of Korea; 2 Department of Food Science and Biotechnology, Ewha Womans University, Seoul 03760, Republic of Korea

**Keywords:** nonylphenols, endocrine disrupting chemical, gas chromatography-mass spectrometry, food

## Abstract

Nonylphenols (NPs) are classified as endocrine-disrupting chemicals (EDCs), which are known to cause disorders in the endocrine systems of organisms. Due to their high lipophilicity and low degradability, these harmful substances are known to accumulate and persist in the environment, and even enter into the food chain. Analytical methods of liquid–liquid extraction using solid-phase extraction for sample clean-up combined with gas chromatography/mass spectrometry were established to determine the presence of NPs in foods. This study aimed to develop and validate these methods using four food matrices representing high-fat and low-fat solid food, as well as high-fat and low-fat liquid food, groups. The single linear isomer 4-n-NP was used to validate the quantification of NPs, which exist in complex isomer mixtures. Our results showed good linearity, with correlation coefficients exceeding 0.998 for all four matrices. The limits of detection and quantification were 0.37–1.79 and 1.11–5.41 μg/kg, respectively. Recovery rates were 86.8–108.6% and 92.6–101.9% for intraday and interday assays, respectively, and the relative standard deviations (RSDs) were below 12% for both assays. The method was applied to analyze 1185 domestic food samples consumed by Koreans, with NPs detected at concentration ranges of 2.57–269.07 μg/kg. Results for each food type over wide concentration ranges indicated that these compounds are highly dependent on the area of cultivation, and are affected by the levels of those contaminants in different environments. The contents of NPs in foods from animal sources were generally higher than those from plant sources, in particular being higher in the intestines than in lean tissue. The present findings could form the basis for determining the level of dietary exposure to NPs and how each food source contributes to it in South Korea.

## 1. Introduction

Nonylphenols (NPs) are persistent and hazardous endocrine-disrupting chemicals (EDCs), which are found in a variety of environmental matrices, including foods, human tissue samples, and blood [1]. EDCs can disrupt hormone biosynthesis, metabolism, or function by causing a disruption in normal homeostasis or reproduction [2]. These compounds interfere with the endocrine system by imitating or suppressing endogenous hormones, resulting in hormonal dysfunction and deleterious effects on living organisms. By competing with natural estrogen for estrogen receptor bindings, NP especially hinders the estrogenic balance in organisms [3]. NPs are a complex mixture of compounds composed of isomers with differently branched carbon side and linear chains, and there can, theoretically, be more than 200 constitutional isomers [4]. Linear 4-n-nonylphenol (4-n-NP) is the least active of the individual isomers, and its relative potency is known to increase with higher levels of nonyl-branching side chains [5]. Different degrading behaviors in these isomer forms of NP have been observed in soil and aquatic environments, with linear NPs degrading more rapidly than branched NPs [6].

Nonylphenol polyethoxylates (NPEOs) are nonionic surfactants that are widely used for detergent, pesticide, and emulsifier production. They are biodegraded into shorter chain compounds and NPs, which do not have ethoxyl groups, under certain conditions [7]. Effluents from sewage treatment plants, industrial discharges, and municipal waste streams are the main sources of these compounds in aquatic ecosystems. During sewage treatment, NPEOs are biodegraded by hydrolytic cleavage of ethoxylate groups into short-chain ethoxylates, and finally to alkylphenols, resulting in the formation of progressively more lipophilic and persistent metabolites. De-ethoxylated NP is more resistant to biodegradation than compounds with long-chain ethoxylates. NPs and NPEOs are persistent in aquatic environments, moderately bioaccumulative, and severely toxic to aquatic organisms, which means that their commercial manufacturing, processing, and distribution pose potential ecological threats.

Humans are exposed to NPs primarily through the intake of contaminated food and water. NPs are particularly known to be present in fish and shellfish due to their exposure to aquatic environments. NPs can also be introduced to soil used for producing crops and grazing livestock when sewage sludge is applied to agricultural land. Through these diverse pathways, NPs remain in the environment, enter the food chain, and finally accumulate in lipid-rich matrices including fish and edible oil [8]. NPs can accumulate in the internal organs of fish, reaching concentrations 10 to 100 times higher than those observed in the environment, due to their strong stability and solubility in lipids. They can then be readily transferred to humans through the food chain. Notably, NP bioaccumulation has been reported in humans, with accumulation values of 57 and 37 ng/g in the adipose tissue of humans and cadaver samples, respectively [9]. NPs were also found in every urine sample examined in a recent investigation conducted in Taiwan, with concentrations ranging from 0.65 to 6.69 ng/mL, demonstrating the human exposure to these types of EDCs [10].

Because of their low cost and high availability, traditional sample treatment techniques with long analysis times and waste production have been used to determine alkylphenols in samples. Conventional procedures, such as Soxhlet extraction, require analysis times from 10 to 48 h [11]. Alkylphenols have also been extracted from food and environmental samples using steam distillation. Despite steam distillation being known to be more simple than Soxhlet extraction, it is also laborious and time-consuming. To develop a simple and efficient method for analyzing NPs, particularly in high-fat food samples, solid-phase extraction (SPE) was combined with liquid–liquid extraction to reduce the isolation time for analytes. Due to its versatility, SPE has been widely used to separate and enrich NPEOs and their degradation products, NPs, from food and environmental matrices. Many SPE procedures have been devised using various sorbents with many variables, such as SPE sorbent and eluent types, potentially influencing the recovery of target compounds [12].

Since there was no literature on monitoring NP contents in foods from Korean markets, it was necessary to determine the concentrations of NPs in various food groups based on national consumption. Previous studies on NPs in foods are also scarce, although one of the major exposure routes to humans was oral food ingestion. The objective of the present study was to develop, optimize, and validate a simple, efficient, and sensitive integrated analysis method to determine NPs in high-fat and low-fat matrices. The established method was further applied to analyze the concentrations of NPs in 1185 food samples categorized as aquatic, livestock, and agricultural products.

## 2. Materials and Methods

### 2.1. Chemicals and Reagents

Nonylphenols (PESTANAL^®^, analytical standard, technical mixture, purity 96.8%) and 4-n-nonylphenol (PESTANAL^®^, analytical standard, purity 99.9%) were purchased from Sigma-Aldrich (Saint Louis, MO, USA). 4-n-nonylphenol RING-^13^C_6_ 100 μg/mL in nonane was purchased from Cambridge Isotope Laboratories (Tewksbury, MA, USA, purity 99.9%). Methylene chloride, acetonitrile, methanol, and hexane were acquired from J.T. Baker (Philipsburg, NJ, USA). Mega Bond Elut-C18 OH (1 g, 6 mL) was purchased from Agilent (Santa Clara, CA, USA). Visiprep™ SPE Vacuum Manifold and disposable liners for SPE Vacuum Manifold were obtained from Supelco (Saint Louis, MO, USA).

### 2.2. Sample Selection and Preparation

The food samples for analysis were selected based on the “National Nutrition Statistics, Food Consumption Distribution” document published by the Korea Health Industry Development Institute (2017). Based on consumption rates and detection levels from previous studies, 79 food categories were chosen. Six major cities in Korea (Seoul, Busan, Incheon, Daegu, Daejeon, and Gwangju) were selected based on the population status data of the local government. Samples were all collected on the same day from marts and markets with high average annual sales in each city. Foods from each category were purchased from 15 different markets nationwide.

Food samples used for monitoring were analyzed in raw conditions, without having received treatments such as cooking or seasoning, and only the edible portions were separated and pretreated. Each sample was treated and homogenized using liquid nitrogen and a blender (Nutri Ninja Auto iQ, Hai Xin Technology), respectively. Each homogenized sample was placed and sealed in a separate HDPE bottle, then stored at –70°C in a deep freezer until the analysis.

### 2.3. Food Matrix Types

Food matrices were divided into high-fat and low-fat groups. The foods representing each matrix were as follows: high-fat solid, infant purée; high-fat liquid, olive oil; low-fat solid, enoki mushroom; and low-fat liquid, 7.00% ethanol solution. These matrices were selected based on the lipid contents from the standard guidelines of the Korean Total Diet Study by the Korean Ministry of Food and Drug Safety. Although 7% ethanol solution was not a real food, it was selected for the matrix of low fat liquid foods. The 7% ethanol content is in the range of commercial liquor products in Korea, which would represent alcoholic beverages. The additional advantage of its use would be less contamination of nonylphenols compared to other food matrices.

### 2.4. Preparation of Standards

A stock solution of 4-n-NP (1.00 × 10^3^ μg/mL) was prepared by dissolving 0.01 g of 4-n-NP using 10.0 mL of methanol. A working solution (100 μg/mL) was prepared with a methanol dilution 10 times higher than the stock solution.

An internal standard 4-n-NP RING-^13^C_6_ (10.0 μg/mL in methanol) was prepared by diluting 1.00 × 10^2^ μg/mL in nonane using methanol. Regarding the standard curve of the low-fat matrix, standard solutions of 4-n-NP were spiked at concentrations of 1.00 × 10^1^, 2.00 × 10^1^, 1.00 × 10^2^, 5.00 × 10^2^, and 1.00 × 10^3^ ng/mL; for the standard curve of the high-fat matrix, standard solutions of 4-n-NP were spiked at concentrations of 1.00 × 10^2^, 2.00 × 10^2^, 5.00 × 10^2^, 1.00 × 10^3^, and 2.00 × 10^3^ ng/mL.

### 2.5. Analysis Methods for Each Matrix

Analytical procedures for low-fat and high-fat matrices were based on the official ES 04613.1 method of the National Institute of Environmental Research (2017) and a modified version of another previous method [13].

For high-fat matrices, a 20.0-μL working solution of 4-n-NP RING-^13^C_6_ was added to 3.00 g of infant purée, along with 9.00 mL of acetonitrile. After vortexing the mixture for 1 min, the mixture was centrifuged at 1977× *g* at 0 °C for 5 min. The acetonitrile phase mixture was then collected. The mixture was extracted again using 9 mL of acetonitrile, as described above. The acetonitrile phase mixture was obtained and combined with the previously collected acetonitrile. After adding Mega Bond Elut-C18 OH combined with disposable liners to the Visiprep™ SPE Vacuum Manifold, the SPE cartridge was activated with 5.00 mL of both methylene chloride and acetonitrile. From this mixture, 3.00 mL of collected acetonitrile was extracted and poured into the activated cartridge. The analyte was eluted with 5.00 mL of acetonitrile. The collected elution was completely evaporated using N_2_ flow, and the residue was redissolved with 0.300 mL of hexane. The procedure described above was used to prepare 3.00 g of olive oil. 

For low-fat matrices, a working solution of 10.0 μL of 4-n-NP RING-^13^C_6_ was added to 3.00 g of enoki mushroom in a separatory funnel. The mixture was added to 20.0 mL of methylene chloride for liquid–liquid extraction. The separatory funnel was agitated vigorously using a vertical shaker for 20 min. After separating the methylene chloride from the mixture, the collected elution was completely evaporated using N_2_ flow. The dried residue was redissolved with 0.300 mL of methanol. The procedure described above was used to prepare 3.00 g of 7.00% ethanol solution.

### 2.6. Evaluation of Procedural Blanks

Due to the widespread usage of NP-containing substances, including consumer goods, NPs are often present in laboratory environments. These environments are also susceptible to contamination from external sources [5]. Both NPs and their parent compounds, NPEOs, are, therefore, ubiquitous in the environment due to the increasing production volumes of NPs in industry [14]. Special precautions are, therefore, required to prevent contamination during experiments [13]. NP residues were regularly present in the blank samples in this study to ensure that these compounds were not contaminated during the experiment procedure. To minimize contamination during the experiment and to properly control the procedural blank, glassware was mostly used throughout the experiments. Procedural blanks were analyzed to evaluate the analytical methods of the high-fat and low-fat matrices. The analytical procedure was identical to that illustrated above, except for the sample addition. Procedural blanks were analyzed using gas chromatography/mass spectrometry (GC-MS) as described below.

### 2.7. Instrument Analysis

The samples were analyzed using a 7890A gas chromatograph with a 5977B mass spectrometer. A DB-5ms capillary column was used for separation. Helium (99.9999% purity) was used as a carrier gas, with a 0.8 mL/min constant flow rate. Injection and ion source temperatures were set at 230 °C. The oven temperature program was as follows: initial temperature of 80 °C, 10 °C/min increase to 180 °C, 3 /min increase to 240 °C, and finally a post run at 250 °C for 15 min. The 1.0-μL sample volume was injected in splitless mode. 

The mass spectrometer was operated in its SIM mode for quantification. The monitored fragment ions had m/z values of 121, 135, 149, and 163 for NP, m/z values of 107 and 220 for 4-n-NP, and m/z values of 113 and 226 for 4-n-NP RING-^13^C_6_.

**Figure 1:**
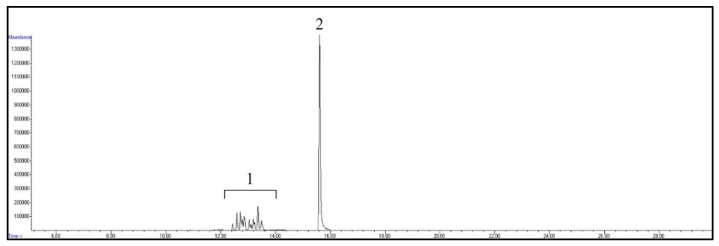
GC/MS total ion chromatogram of analytes. Peaks: 1, NPs; 2, 4-n-NP and 4-n-NP ^13^C_6_.

**Figure 2:**
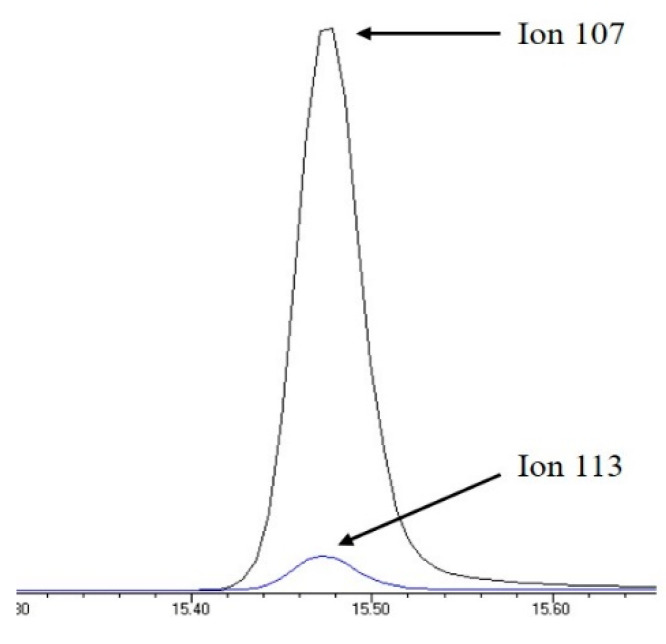
Selected ion chromatogram of quantifier ions for 4-n-NP and 4-n-NP ^13^C_6_.

The retention time and the qualifier and quantifier ions of each compound are listed in Table 1. The total ion chromatogram of analytes and the chromatogram of selected ions for 4-n-NP and 4-n-NP ^13^C_6_ are shown in Figure 1 and Figure 2, respectively.

### 2.8. Validation Method

NPs are frequently regarded as a single compound when their environmental occurrence, transit, removal, and toxicity are investigated. However, technical NPs comprise a mixture of over 100 isomers and congeners with different side chain lengths and branches [15]. The NP chromatogram presents more than 10 isomer peaks with various nonyl substituent branching configurations. Due to the characteristic of NPs being present in more than 100 isomers, some of the individual peaks can contain several isomers and are, therefore, not pure compounds. Furthermore, their compositions vary between manufacturers, and the concentration and exact type of each isomer are unknown [16]. The concentration and type for each isomer in standard branched NP chemicals used for the present investigation were also not identified.

Individual NPs are not commercially accessible, and the standard chemical is a mixture of isomers with alkyl groups with varying branches. Validation for the analysis and calibration curve, therefore, proceeded with 4-n-NP, a single linear compound whose concentration can be accurately determined, instead of branched NPs. The proposed analytical method was validated in terms of its linearity, accuracy, precision, and repeatability. 

For high-fat matrices, the linearity of the calibration curve was determined at five points (*n* = 3), with concentrations of 4-n-NP ranging from 1.00 × 10^2^ to 2.00 × 10^3^ ng/mL at five points (1.00 × 10^2^, 2.00 × 10^2^, 5.00 × 10^2^, 1.00 × 10^3^, and 2.00 × 10^3^ ng/mL), while for low-fat matrices, it ranged from 1.00 × 10^1^ to 1.00 × 10^3^ ng/mL at five points (1.00 × 10^1^, 2.00 × 10^1^, 1.00 × 10^2^, 5.00 × 10^2^, and 1.00 × 10^3^ ng/mL). A calibration curve was obtained using the peak area and concentration ratio of 4-n-NP to 4-n-NP ^13^C_6_.

To determine the sensitivity of the method, the limit of detection (LOD) and limit of quantification (LOQ) were determined as follows:

LOD = 3.3σ/S LOQ = 10σ/S

where S is the standard curve slope and σ is the RSD of the y-intercept.

Accuracy and precision were calculated as a percentage of spiked 4-n-NP in each matrix, and then compared with the AOAC guideline standard value (AOAC, 2012). Internal standards as well as 4-n-NP at three different concentrations were added to each sample. Intraday and interday precision and accuracy were validated by determining analytes in three replicates at the nominal concentrations on three consecutive days. The nominal concentrations were 5.00 × 10^2^, 1.00 × 10^3^, and 2.00 × 10^3^ ng/mL for the high-fat matrices, and 1.00 × 10^2^, 5.00 × 10^2^, and 1.00 × 10^3^ ng/mL for the low-fat matrices. Precision was calculated as the RSD by dividing the standard deviation by the mean, while measurement accuracy was determined by expressing the calculated concentration as a percentage of the nominal concentration.

### 2.9. Identification and Quantification of Branched NPs

NPs were identified by their retention times, and the quantifier and qualifier ions by standard chemicals. Branched NP concentrations were obtained by summing the isomers. For NP quantification in 1185 food samples, the calibration curve obtained using 4-n-NP and relative detected responses of linear and branched compounds was used. The relative response factor was calculated with respect to the area of corresponding analytes with equivalent amounts, in order to convert the concentration obtained using linear 4-n-NP into the total concentration of branched NP isomers. The recovery rates for conversion from 4-n-NP to NPs, which are both composed of isomers, were assumed to be identical, and only detected response values were considered [17].

### 2.10. Statistical Analysis

All experiments were performed once per sample without replications, and thus, we were unable show reproducibility and precision in the analysis of the samples, although our analytical method was validated as explained earlier. The values of NPs were presented as average ± standard deviation of independent data from 15 different samples for each food type. SPSS (version 12.0, Chicago, IL, USA) was used for statistical results.

## 3. Results and Discussion

### 3.1. Method Validation

The linearity of each matrix was verified at five 4-n-NP different concentrations. All four matrices had correlation coefficients exceeding 0.998. The LOD and LOQ values for 4-n-NP were 0.37–1.79 and 1.11–5.41 μg/kg, respectively. The low-fat matrices had lower LOD and LOQ values than the high-fat matrices. The LOD and LOQ values were comparable to or lower than those in the corresponding matrices of previous investigations, with values of 0.83 and 2.5 μg/kg, respectively, for vegetable oils, 1.3 and 4.4 μg/kg for baby food, 1.3 and 3.9 μg/kg for beverages, and 0.3 and 1.0 μg/kg for vegetables and fruits [2]. Table 2 lists the results for linearity, LOD, and LOQ.

The accuracies of the high-fat matrices were 91.8–108.6% and 94.6–101.9% for intraday and interday assays of 4-n-NP, respectively; the corresponding values for the low-fat matrices were 86.8–107.3% and 92.6–101.3%. The accuracies of the high-fat matrices were 1.80–9.68% and 5.42–6.43% for intraday and interday assays of 4-n-NP, respectively; the corresponding values for the low-fat matrices were 0.67–11.83% and 1.65–10.45%. Table 3 lists the results for accuracy and precision.

### 3.2. Procedural Blanks

Procedural blanks were monitored regularly to inspect NP contamination sources during the experiments. The blank values for all matrices were meant to be lower than detectable levels for each matrix. In this study, the results of the procedural blank evaluation demonstrated that the blank NP values were all below the detection limit of the method.

### 3.3. NP Monitoring in Diverse Food Samples

The established analysis method was applied to 1185 food samples to determine NP concentrations. Results for each food group are listed in Table 4. NP contents appeared to be higher in aquatic products than in other food groups, such as agricultural and livestock products. Due to their lipophilic and bioaccumulative properties, alkylphenols remain in the fat tissue of animals throughout the food chain [8]. The NP levels of foods from animal sources were found to be higher than those from vegetables and fruits in Taiwan [8].

NP concentrations varied between species (Tables S1–S3). The concentration ranges for all aquatic products were wide, and the standard deviations were large even within the same food type. Many factors are expected to affect this heterogeneity of the NPs, including feeding patterns and metabolism, degrees of pollution levels in specific habitats, biotransformation, and excretion capabilities. Among these various factors, the habitat environments of each aquatic organism seemed to be a major cause of NP contamination. For example, eels collected from three distinct areas of lagoons and lakes in Poland had varying NP levels, demonstrating that NP concentrations in fish were highly dependent on the sampling area [18]. The alkylphenol concentrations in fish intestines were also closely related to how close sampling sites were to sewage treatment plants. The first sampling site downstream of a treatment plant had the highest NP levels, which declined as the distance from the plant increased. The number of samples was limited, and they were pretreated as pooled samples rather than individual liver samples, making specific correlations with environmental data unclear. However, NP concentrations appeared to follow a similar pattern as the ambient data [19]. Furthermore, different species of wild freshwater and marine fish from the same river or harbor had varying NP concentrations, implying that the potential of fish species for NP bioaccumulation may depend on habitat conditions, the metabolic activity of each aquatic organism, and their feeding strategy [20].

NP concentrations in blue crabs, kelps, and seaweeds among marine algae were found to be relatively high, with mean levels around or higher than 100 µg/kg. Blue crabs, in particular, are known to be inhabitants of benthic environments and sediment dwellers, making them vulnerable to estrogenic chemicals that are substantially concentrated in the sediments below the water [21]. In sediment samples of the coast of Hong Kong, NPs were found in higher concentrations than in water samples, with average values ranging from 3–8 times higher [22]. Because these species experience direct contact with sediments, they are likely to absorb a considerable amount of NPs. In kelps and seaweeds, NPs would be enriched due to the absorption of these compounds, which is a similar tendency to the concentration of seaweed samples from China [23]. 

**Figure 3:**
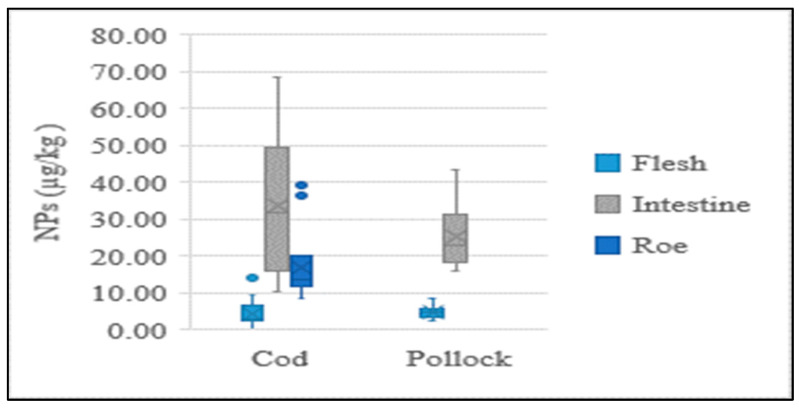
Average concentration of NPs in flesh, intestine, and roe of fish.

The present study performed NP analysis for each individual part of the cod and pollock fish, which revealed different concentrations. The NP contents in the intestines and eggs of cod and pollock were significantly higher than those in their flesh. The concentration distributions of each sample type are shown in Figure 3. A recent study found that the average content in flounder livers was 10.7 times higher than that in flounder muscles, while it was 4.3~19.1 times higher in eel livers than in eel muscles [18], which was a similar tendency to that found in the present study.

Our results indicate that NPs in fish flesh, which is primarily composed of muscles, are more stable and less varied than in the intestines, suggesting that these chemicals do not readily metabolize in the muscles. Low concentrations in the flesh may be attributed to their effective absorption and elimination by the liver and kidneys, as well as a lack of blood flow to the muscles. A smaller deviation in the concentrations of these compounds was observed in the flesh, which possibly indicates that NPs in fish muscles mostly come from the water. Chemical absorption from water is constant and unaffected by the diet or the availability of food for the fish. Because NPs are pollutants metabolized by fish, their levels in tissues vary depending on various factors, such as the routes and metabolic rates of NPs and the reproductive maturity and feeding behaviors of the fish [18]. This makes comparisons of the levels of these compounds between fish species or fish captured in different aquatic basins challenging.

In a previous study, the total concentration of NP compounds analyzed in freshwater fish from other contaminated sites was as high as 5 µg/g (wet), while NPs in carps and yellow perches ranged from 18 to 2075 ng/g (wet). The presence of high quantities of NP compounds in fish suggest that they are persistent, hydrophobic, and bioaccumulative in aquatic organisms [24]. Once NPEOs enter the sediment, their degradation half-life is estimated to be approximately 60 years [25]. The presence of NPs in the environment will, therefore, continue to affect living organisms, which will consequently remain in the food chain as people consume food.

For livestock products, two key factors are expected to contribute to contamination of NPs: (1) bioaccumulation of compounds throughout the food chain and (2) migration from plastics used for packaging food products. The NP concentrations differed between the types of livestock and poultry products, and the differences were relatively large even within the same food type. Exposure to NPs of animals would mostly occur from food consumption. The amount of these compounds will, therefore, inevitably differ due to variations in the types and amounts of crops which each animal ingests. Furthermore, due to pollutant biomagnification in the food web, animals at the top of the chain are thought to have high levels of exposure to xenobiotics [26]. These chemicals are ingested mostly through their diets, especially for ruminants. A large proportion of cellulose can be found in crop residue and agroindustrial by-products such as straw in cereal and maize stover. These fibrous by-products are difficult for the rumens of ruminants to break down. Several feed additives capable of altering the fiber fermentation and digestion of ruminants are produced to enhance the ruminal environment for the purpose of promoting the efficiency of consuming roughage. As a result, nonionic surfactants can be used as feed additives for livestock. Tween 80, which contains NPEOs, is an example of a typical feed additive [27]. Our results indicated that beef had the highest average NP concentration among the analyzed livestock products. Since cows were the only ruminant in the livestock products analyzed in the present study, using NPs as feed additives was likely related to their high beef content.

Another route of NP contamination in foods is their packaging process. From plastic packaging materials in which NPs are added as antioxidants, for example, tris(nonylphenol) phosphate could migrate into foods [28]. Kawamura et al. investigated how much PVC stretch film migrated into fatty and nonfatty food types, and found that NPs moved into fatty foods at a higher rate than into nonfatty foods due to their lipophilic properties [29].

**Figure 4:**
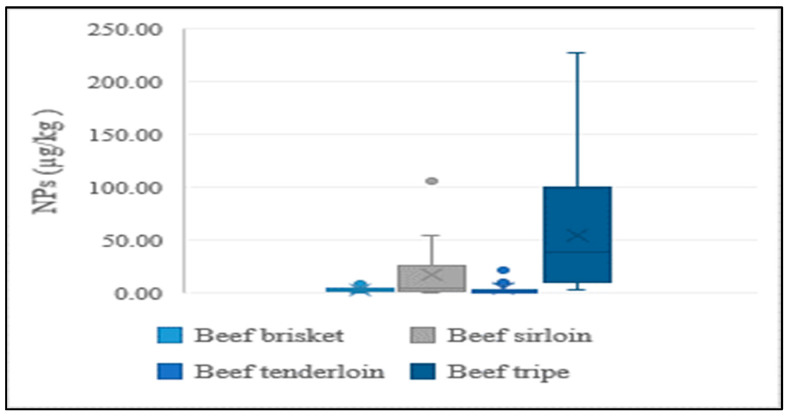
Average concentration of NPs in different edible parts of beef.

**Figure 5:**
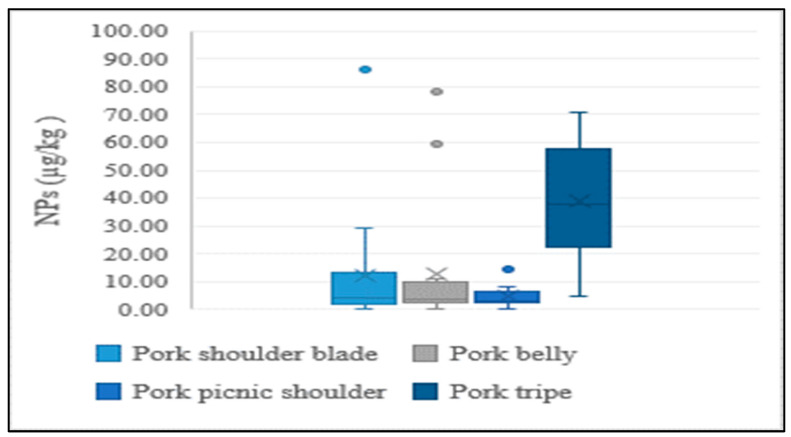
Average concentration of NPs in different edible parts of pork.

In addition to the lean meat portions of livestock, the intestines of cattle and pigs were also analyzed in the present study, considering their high rate of consumption by Koreans. NP levels were higher in intestines than in lean tissue, indicating a similar tendency to aquatic products. This could be explained by NPs having a stronger and greater affinity to tissues than intestines. Results of the distribution of concentrations for different edible portions of livestock are presented in Figure 4 and Figure 5.

No previous study has compared the NP levels in the intestines and lean tissue of livestock. However, different internal tissues of birds, including ducks, were found to have a specific affinity to NPs. NPs accumulated the most in the muscles of each bird species, followed by the livers and kidneys. The various levels that accumulated in their livers and kidneys could possibly reflect differing degrees of excretion from the body due to a varied affinity to fatty tissues [26]. That tendency for bird intestines to accumulate NPs is expected to occur similarly in livestock products. When NPs are consumed through food or water, the intestine acts as a first barrier, absorbing and glucuronidating them. However, because the alkylphenol transport mechanism is hindered by its long alkyl chain, NP removal from the intestine is delayed, resulting in its accumulation in intestinal tissue. This NP accumulation causes its steady release into the bloodstream, which eventually reaches the liver. Although the liver can metabolize NPs, it also has a transport system that is restricted by long alkyl groups [30].

The lipid contents and NP concentrations of chicken and duck meat are listed in Table 5. Chicken breast had the lowest NP level, as well as the lowest lipid content, among types of chicken meat, with other portions with larger lipid contents having six-fold to nine-fold higher average NP levels. Additionally, the highest average NP concentration among poultry products was found in duck meat, which had the highest lipid content, possibly due to the aquatic environment being contaminated, and ducks being an aquicolous species [31].

**Figure 6:**
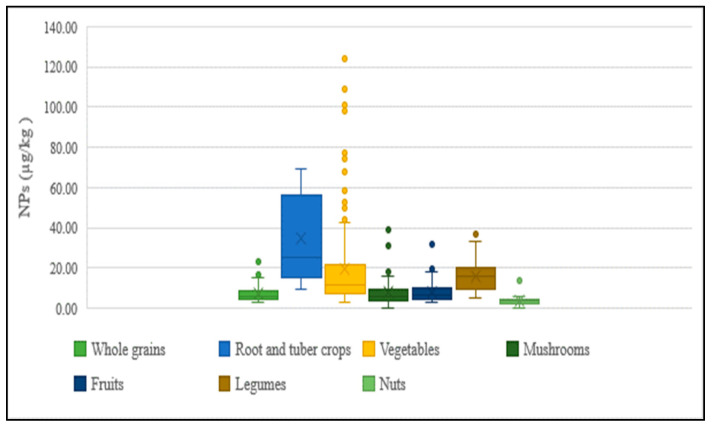
Concentration of NPs in agricultural products.

The presence of NPs in agricultural products purchased from markets has been observed to vary between regions, with its concentration differing between and within vegetable species. The distribution of concentrations for each food category of agricultural products is shown in Figure 6. The main factors expected to contribute to this large variation are as follows: the differences in the degree of contamination of sludge and soil used for cultivation, and the application of pesticide products containing varying amounts of NPEOs during cultivation.

Applying sludge to agricultural land is a cost-effective technique for sewage disposal, and is one of the most common methods of sludge management. NPEOs are biodegraded in sewage treatment by the hydrolysis of ethoxylate groups, producing short-chain ethoxylates and NPs. Significant amounts of these breakdown products have been found in sewage effluents, surface waters, and sediments. Accumulation in sewage sludge induced the release of high NP concentrations into the environment once the sludge is applied to agricultural land. NPs may contact crops after being applied to soils, where they might be absorbed and accumulated through their root systems. A greater NP persistence in soil means a higher potential for crop uptake [32]. Furthermore, because of its hydrophobic property, NPs adsorb into solid organic particles in soil, and eventually bioaccumulate over time to produce large amounts [33].

NP breakdown in soil and water is influenced by factors such as oxygen availability and microbial activity, resulting in observations of substantial differences in NP persistence. The degradation of long-chain NPEOs begins with ethoxylate chains being cleaved into short chains, such as NP1EO and NP2EO. These short-chain ethoxylates further degrade into NPs, indicating that NPs are likely to accumulate due to polyethoxylate breakdown. The physical, chemical, and biological properties of individual soils ultimately control NP degradation and plant availability [32]. These results suggest that NP concentration depends on the soil where crops are grown, which can have a wide range even within the same food type of agricultural products.

Another possible route for NPs reaching agricultural products is through adjuvants in pesticides. NPEOs are commonly used as nonionic surfactants in pesticide formulations due to their low cost and high performance. These surfactants are used in agricultural pesticides for various reasons, including increasing droplet spreading, lowering surface tension, lowering solvent evaporation rates, increasing residence duration on plant surfaces, and improving pesticide suspension and emulsion stability [33]. Using pesticides and fertilizers in agriculture can lead to NPEO degradation product accumulation with shorter-chain ethoxylate groups, such as NPs, on crop surfaces. Ethoxylate degradation to NPs could result in NP accumulation in fruits, vegetables, and cereals treated with pesticides. Furthermore, NPs can remain in the soil for months after sludge application, resulting in its uptake in plants [34]. NP residues are particularly shown to be primarily generated by pesticides used in the cultivation of leafy vegetables. High NP concentrations were observed in commercial pesticide products commonly used in China, significantly differing within a range of 138~1245 mg/kg [35].

Due to the varied concentrations of these compounds in different vegetables and crops, most samples were considered to be contaminated by NPs via diverse pathways at various stages of the food production process. Some of this contamination would derive from alkylphenol ethoxylates, which are utilized in disinfectants and pesticide formulations as nonionic surfactants and emulsifiers. NPEO degradation products could induce NP accumulation on the roots and other portions of vegetables and crops after being used in agriculture. Another possible source is degradation products such as NP residues from tris(nonylphenol) phosphate, a component of plastic, which could migrate into vegetables when applied by methods involving food contact [7]. Tris(nonylphenol) phosphate is produced when NPs react with phosphorous trichloride, which is applied as an antioxidant stabilizer. As a result, when the plastic is utilized in ways where it contacts food, NP residue degradation products or impurities from tris(nonylphenol) phosphate in the plastic may migrate into agricultural products [36].

## 4. Conclusions

Analytical methods were developed and validated for four different matrices to determine the NP concentrations in various foods. The linearity, sensitivity, accuracy, and precision results demonstrated that the methods established in this study are efficient for determining analytes in four different matrices with varied food compositions. 

The results of the analyzed NPs in foods indicate that these compounds are ubiquitous in food products in Korea, suggesting the possibility of disrupting the endocrine systems of organisms. The monitoring of these substances should, therefore, be updated regularly in order to evaluate dietary exposure in humans. The separation and identification of branched NPs in a single run still represent a great challenge due to the coexistence of many different NP isomers. Further research should, therefore, be conducted, with a focus on increasing the separation of these isomers using sophisticated analytical methods, such as GC-MS/MS and GCxGC/MS.

## Figures and Tables

**Table 1:** Mass spectral parameter of the analyzed compounds.

Compound	Abbreviation	Retention Times	Qualifier Ions	Quantifier Ions
Nonylphenol	NP	12.0–13.7	121, 149, 163	135
4-n-nonylphenol	4-n-NP	15.6	220	107
4-n-nonylphenol ^13^C_6_ *	4-n-NP ^13^C_6_	15.6	226	113

* Internal Standard.

**Table 2:** Linearity, LOD, and LOQ for 4-n-NP in food matrices.

Food Type	Matrix	Standard Curve	Linearity (R^2^)	LOD (μg/kg)	LOQ (μg/kg)
High-fat	Infant puree	y = 2.85 x + 1.72 × 10^−1^	9.98 × 10^−1^	1.79	5.41
	Olive oil	y = 2.95 x + 8.98 × 10^−3^	9.99 × 10^−1^	6.39 × 10^−1^	1.94
Low-fat	Enoki mushroom	y = 1.96 x + 5.04 × 10^−2^	9.98 × 10^−1^	3.66 × 10^−1^	1.11
	7% ethanol	y = 2.31 x + 8.86 × 10^−2^	9.99 × 10^−1^	3.82 × 10^−1^	1.16

**Table 3:** Accuracy and precision for 4-n-NP in four food matrices (*n* = 3).

Matrix	Nominal Concentration (ng/mL)	Intraday 1		Intraday 2		Intraday 3		Interday	
		Accuracy (%)	Precision (% RSD)	Accuracy (%)	Precision (% RSD)	Accuracy (%)	Precision (% RSD)	Accuracy (%)	Precision (% RSD)
Puree	500	97.2	7.68	92.8	7.19	93.9	6.05	94.6	6.43
	1000	95.9	4.12	93.0	4.95	101.0	5.23	96.6	5.53
	2000	103.7	7.16	97.7	4.07	104.2	3.41	101.9	5.42
Olive oil	500	97.1	9.68	91.8	4.25	97.7	2.51	95.5	6.23
	1000	95.4	2.00	97.7	7.72	99.8	7.10	97.6	5.74
	2000	98.0	2.64	108.6	1.80	97.4	2.98	101.3	5.80
Enoki mushroom	100	102.5	11.32	88.7	0.67	86.8	6.44	92.6	10.45
	500	107.3	6.45	99.8	11.83	93.9	6.12	100.3	9.36
	1000	92.9	5.88	97.9	8.19	103.3	4.68	98.0	7.16
7% EtOH	100	100.5	2.08	100.7	1.76	99.1	1.14	100.1	1.65
	500	102.8	2.72	99.3	4.60	101.9	3.80	101.3	3.61
	1000	100.3	4.30	99.6	1.50	101.0	1.63	100.3	2.49

**Table 4:** Concentration of NPs in food groups.

Food Category	NPs (µg/kg)	
	Mean	Min~Max
Shellfish	21.27	N.D. *~72.80
Cephalopods	4.65	N.D.~41.89
Crustaceans	58.64	N.D.~252.65
Fresh water and saltwater fish	11.44	N.D.~68.78
Marine algae *	75.62	N.D.~269.07
Poultry	14.02	N.D.~50.59
Livestock	18.36	N.D.~227.06
Eggs	2.17	N.D.~7.36
Oils and fats	0.15	N.D.~1.14
Whole grains	7.34	3.26~23.08
Legumes	16.28	5.32~36.58
Root and tuber crops	34.70	9.54~69.27
Vegetables	19.88	2.68~123.91
Mushrooms	7.88	N.D.~39.22
Fruits	7.92	3.17~31.68
Nuts	3.82	N.D.~14.30

* N.D.: lower than the limit of detection.

**Table 5:** Lipid contents and NP concentrations for chicken and duck meat.

Food Samples	Lipid Contents (%) *	Average NP Level (µg/kg)
Chicken wings	10.5	13.0
Chicken breast	1.00	2.17
Chicken legs	7.70	19.8
Duck	21.0	21.1

* Lipid contents were referred to the food nutrition database of the Ministry of Food and Drug Safety (2019).

## Data Availability

Data are contained within the article.

## Supplementary Materials

The following supporting information can be downloaded at: https://www.mdpi.com/article/10.3390/foods12020269/s1, Table S1: Concentration of NPs in aquatic products; Table S2: Concentration of NPs in livestock products; Table S3: Concentration of NPs in agricultural product.
