# Supplementary material for: Analysis of Endocrine Disrupting Nonylphenols in Foods by Gas Chromatography-Mass Spectrometry
Source: Foods. 2023 Jan 6;12(2):269. doi: 10.3390/foods12020269 (PMC9858244; doi:10.3390/foods12020269)
Supplement: Supplementary file 1 [file foods-12-00269-s001.zip › foods-2116352-supplementary.pdf]

**Table S1.** Concentration of NPs in aquatic products (N=15).

| Food category | NPs(μg/kg) | |
|---|---|---|
| | **Mean±SD** | **Min~Max** |
| **Shellfish** | | |
| Oyster | 25.98±21.16 | 5.45~69.74 |
| Manila clam | 13.65±13.87 | 2.39~45.74 |
| Cockle | 25.79±16.66 | 7.33~62.12 |
| Common orient clam | 10.86±8.65 | 2.65~29.11 |
| Mussel | 37.04±16.89 | 14.04~72.80 |
| Whelk | 13.68±11.46 | N.D.[a]~32.39 |
| **Cephalopods** | | |
| Squid | 8.57±10.80 | 2.00~41.89 |
| Octopus | 2.12±2.66 | N.D.~10.28 |
| Poulp | 3.26±1.63 | N.D.~7.63 |
| **Crustaceans** | | |
| Shrimp | 4.54±3.80 | N.D.~16.95 |
| Blue crab | 112.75±83.07 | 11.03~252.65 |
| **Fresh water and saltwater fish** | | |
| Salmon | 6.39±9.04 | N.D.~32.31 |
| Cod flesh | 4.52±3.50 | N.D.~13.91 |
| Cod intestines | 33.50±18.08 | 10.16~68.78 |
| Cod roe | 17.05±9.07 | 8.72~39.21 |
| Mackerel | 2.78±3.16 | N.D.~10.52 |
| Pomfret | 6.82±4.35 | N.D.~15.05 |
| Sea bass | 7.33±4.84 | 2.46~17.43 |
| Pollock flesh | 4.83±1.74 | 2.56~8.58 |
| Pollock intestines | 25.36±8.46 | 16.02~43.27 |
| Flatfish | 15.80±21.29 | N.D.~53.25 |
| Hairtail fish | 7.29±7.13 | N.D.~26.51 |
| Pacific saury | 19.44±14.02 | 3.60~48.78 |
| Anchovy | 17.75±12.80 | 3.92~41.92 |
| Japanese Spanish mackerel | 4.41±3.66 | N.D.~12.44 |
| Tuna | 3.32±2.70 | N.D.~8.00 |
| Eel | 6.46±8.58 | N.D.~31.67 |
| **Marine algae** | | |
| Kelp | 76.39±24.57 | 35.36~118.06 |
| Laver | 8.62±3.74 | N.D.~13.56 |
| Seaweed | 141.85±71.09 | 42.87~269.07 |

[a]N.D.: lower than the limit of detection. *Processed foods

**Table S2.** Concentration of NPs in livestock products (N=15).

| Food category | NPs(μg/kg) | |
|---|---|---|
| | **Mean±SD** | **Min~Max** |
| **Poultry** | | |
| Chicken wings | 13.01±9.59 | 3.17~32.95 |
| Chicken breast | 2.17±1.98 | N.D. [a]~6.84 |
| Chicken legs | 19.81±12.49 | N.D.~38.91 |
| Duck | 21.09±16.79 | 2.00~50.59 |

| | | |
|---|---|---|
| **Livestock** | | |
| Beef brisket | 2.96±2.30 | N.D.~9.13 |
| Beef sirloin | 17.62±29.22 | N.D.~105.64 |
| Beef tenderloin | 3.99±5.73 | N.D.~21.51 |
| Beef tripe | 54.26±60.30 | 2.57~227.06 |
| Pork shoulder blade | 12.07±22.06 | N.D.~86.32 |
| Pork belly | 12.50±23.39 | N.D.~78.23 |
| Pork picnic shoulder | 4.43±3.65 | N.D.~14.48 |
| Pork tripe | 39.03±19.82 | 4.62~70.89 |
| **Eggs** | | |
| Chicken eggs | 1.84±1.80 | N.D.~5.34 |
| Quail eggs | 2.50±2.25 | N.D.~7.36 |
| **Oils and fats** | | |
| Soybean oil* | N.D. | N.D. |
| Sesame oil* | 0.45±0.45 | N.D.~1.14 |
| Butter* | N.D. | N.D. |

[a]N.D.: lower than the limit of detection. *Processed foods

**Table S3.** Concentration of NPs in agricultural products (N=15).

| Food category | NPs(μg/kg) | |
|---|---|---|
| | **Mean±SD** | **Min~Max** |
| **Whole grains** | | |
| Flour* | 5.22±1.98 | 3.88~11.40 |
| Brown rice | 7.75±1.99 | 5.00~11.74 |
| Rice | 3.99±0.97 | 3.26~7.09 |
| Corn | 8.62±5.80 | 4.09~23.08 |
| Oats | 11.14±3.98 | 4.74~18.75 |
| **Legumes** | | |
| Soybean | 21.48±8.94 | 6.77~36.58 |
| Kidney beans | 9.22±3.39 | 5.32~16.37 |
| Black beans | 18.14±5.22 | 7.24~27.91 |
| **Root and tuber crops** | | |
| Potato | 15.68±5.00 | 9.54~25.52 |
| Sweet potato | 53.73±13.26 | 16.30~69.27 |
| **Vegetables** | | |
| Cucumber | 74.61±32.11 | 10.00~123.91 |
| Watermelon | 30.75±12.83 | 16.53~59.35 |
| Tomato | 17.56±8.37 | 10.53~41.98 |
| Cabbage | 32.36±12.61 | 17.66~58.45 |
| Napa cabbage | 18.06±12.39 | 4.62~41.27 |
| Broccoli | 13.56±5.57 | 6.51~23.52 |
| Cauliflower | 9.91±3.69 | 3.35~14.62 |
| Lettuce | 7.21±3.33 | 2.68~14.45 |
| Spinach | 7.77±3.79 | 3.72~18.03 |
| Onion | 5.45±1.52 | 3.64~8.23 |
| Carrot | 11.13±4.75 | 6.45~20.53 |
| Green onion | 10.17±3.30 | 6.27~16.70 |
| **Mushrooms** | | |
| Shiitake mushroom | 9.97±13.74 | N.D.[a]~39.22 |
| Button mushroom | 4.48±0.82 | 3.49~6.22 |

| | | |
|---|---|---|
| Enoki mushroom | 9.18±2.71 | 5.20~15.88 |
| **Fruits** | | |
| Pineapple | 7.68±2.29 | 4.57~11.97 |
| Banana | 9.07±4.10 | 4.40~18.02 |
| Apple | 8.81±7.49 | 3.74~31.68 |
| Pear | 10.43±2.50 | 6.82~15.16 |
| Grape | 3.59±0.40 | 3.17~4.87 |
| **Nuts** | | |
| Peanut | 2.84±1.38 | N.D.~4.43 |
| Almond | 4.79±3.92 | 1.85~14.30 |

[a]N.D.: lower than the limit of detection
[*]Processed foods
